# Effect of Different Environments’ Conditioning on the Debonding Phenomenon in Fiber-Reinforced Cementitious Matrix-Concrete Joints

**DOI:** 10.3390/ma14247566

**Published:** 2021-12-09

**Authors:** Salvatore Verre

**Affiliations:** Department of Civil Engineering, University of Calabria, 87036 Rende, Italy; salvatore.verre@unical.it; Tel.: +39-0984-494055

**Keywords:** fiber/matrix bond, alkaline environment, hot water environment, durability FRCM-concrete

## Abstract

This paper presents the results of an experimental study conducted to understand the bond capacity through single-lap, direct-shear tests of fiber-reinforced cementitious matrix (FRCM)-concrete joints under an alkaline and hot water environment. The experimental campaign was focused on a FRCM system equipped with two different types of fibers, (PBO) and Carbon. After the conditioning, the specimens conditioned were subjected to visual inspection, and the experimental results were compared with the unconditioned specimens. Moreover, in this present work, the number of layers and the conditioning time were varied.

## 1. Introduction

In recent years, the quest for load-carrying capacity increase in existing structural elements under static and seismic actions has played an impotent role in the use of composite materials. The light weight of composite materials and the ease of application have favored their diffusion all over the world. The first composite material used was called fiber-reinforced polymers (FRP). This FRP reinforcement system consisted of multi-axial fabric meshes immersed in epoxy resin (adhesive) [1,2,3]. A few decades later, a new composite material, called fiber-reinforced cementitious matrix (FRCM) or textile-reinforced mortar (TRM), began to be tested and used. The FRCM system also uses, as in the FRP system, a fabric mesh but is immersed in a cementitious-type matrix (adhesive) for the concrete substrate or lime for the tuff/masonry substrate [4,5,6,7,8,9]. The type of fabric used is of a different natural derivation such as basalt, hemp, and flax fibers or artificial nature such as carbon, glass, steel, and polyparaphenylenebenzobisoxazole (PBO) fibers. Moreover, the FRCM system, due to its intrinsic nature, presents characteristics such as (1) high resistance to fire and high temperature, (2) resistance to UV radiation, and (3) permeability and compatibility with the substrate. FRCM systems base their mechanical behavior and their effectiveness on physical interaction for the stress transfer to the substrate. The bond stress mechanism, as reported in [10,11,12,13,14,15,16,17], is very complex.

Many phenomena are involved between single fibers and matrix: the penetration of the matrix into a bundle and the capacity of wetting single filaments, the bond between external and internal fibers, the non-uniform distribution of the stress in the roving fibers, and the arrangement of the fibers in the fabric mesh. If the reinforcement system is exposed to degradation mechanisms, such as frost and thaw, high temperature, and the attack of soluble salts’ sulfates, chlorides, and carbonates through artificial aging, environments may cause different stress mechanisms. The phenomenon of durability was little investigated, but it is of fundamental importance on the long-term effects of the effectiveness of external reinforcement on reinforced structures. Research reported in [18] focused on the experimental campaign, which was composed of masonry joints equipped with strips of steel-FRCM subjected to artificial weathering cycles in a solution of sodium sulfate decahydrate and sodium chloride. The effects of water-wet and salt-dry caused a decrease of the bond capacity by 26–30%, respectively. While, in terms of failure mode, a failure similar to reference specimens occurred between the steel-fiber and the matrix (intermediate failure), was observed, in some cases, the steel-fiber ruptured.

On the external strip and on the masonry joint, the solution adopted produced an efflorescence. Another weathering cycle was composed of deionized water and was studied in [19,20], in terms of flexural and bond capacity. In both studies, an increase of the capacity was evaluated by means of higher peak load and initial stiffness to the reference specimens. The conditioning time involved the specimens for a few days. The study conducted in [21] on the mechanical behavior of the lime-based FRCM system reinforced with glass fabric immersed in a tap water and saline solution showed a decrease of about of 20% and 29%, respectively, of the bond capacity. Both the research by Donnini et al. [21,22] and Nobili [23] studied the effect of saline, alkaline, and freeze–thaw cycle on FRCM specimens equipped with glass fiber and the single components of dry fiber and mortar. The freeze–thaw cycle, in terms of flexural tensile and compressive strength, induced a slight increment. The alkaline and saline cycle caused a decrease of 50% on the mechanical characteristic of the lime-based mortar, while, in terms of tensile strength of the FRCM specimen, it showed a decrease equal to 10% and 15%, respectively. A void and swelling at the center of the glass fiber were also observed in [22]. Other important aspects are the time of curing and environmental exposure. In [23,24], the effects of saline and alkaline environments on FRCM specimens equipped with carbon fiber with different cure times of 28 and 60 days, respectively, were also studied. The results obtained for the 28-day and the 60-day curing were similar. However, with a longer curing time, it was possible to note a great mitigating effect on the impact of the aggressive environment with a beneficial effect close to 30% of the unexposed value.

In the research conducted by Micelli and Aiello in [24], the experimental campaign was focused on the residual mechanical tensile properties of different dry and impregnation fibers, after the exposure to four different aging protocols in alkaline solution, and the bond strength on different dry fibers exposed to alkaline environment. The fibers investigated were different types of glass, basalt, aramid, carbon, and steel. The glass and basalt fiber showed a high sensitivity to the alkaline environment, with a decrease in terms of strength equal to 86% having a 6-month exposure. Instead, the aramid fiber showed a higher chemical resistance to the alkaline environment with a decrement equal to 20%. The steel and carbon fibers did not exhibit effects after the alkaline exposure. In [25], an extensive study was conducted on the FRCM system equipped with PBO and carbon fiber types under different environmental conditions (frost and chemical attack) in order to evaluate the durability characteristics. The conditioned environment did not produce degradation to the FRCM specimens; in a few cases, the peak stress was higher than the reference stresses (control), but the different conditioning induced different failure modes. Finally, in [26], a review was reported on all tests conducted on FRCM systems. The conditioned environments considered were freeze–thaw cycles, hygrothermal, and concentrated solution (chloride attack, seawater, and sulfate attack) in order to evaluate the FRCM behavior in the long term, while the test types were related to the specimen types in order to evaluate the mechanical characteristics. In particular, for the mortar, the flexural tensile and compressive strength were considered. On the single bundles and dry fibers, the tensile test with different guidelines [27,28] was considered; the same test type was applied on the FRCM specimen. Finally, the bond strength was evaluated through a single-lap, direct-shear test. The principal aspects investigated in this experimental campaign were the effects of the alkaline environment on the mechanical characteristics and the stress-transfer mechanism between the fiber and the matrix and the composite strip and the concrete substrate. The parameters investigated were the number of layers, FRCM reinforcement type, and type of conditioning. In particular, the tests were performed through the main techniques as tensile test and the single-lap, direct-shear test.

## 2. Experimental Investigation

Many studies on the bond between FRCM strengthening systems and concrete joint have been carried out. The obtained results are able to define a first step in understanding the macro-aspects/effects of the durability related to the FRCM-concrete bond mechanisms. However, few studies are present in the literature on the effect of the durability between the FRCM system and the concrete joints. The effects of artificial-aging environments are poorly investigated for the mechanical properties of FRCMs and interaction with the concrete substrate. The aim of the experimental result presented and discussed in this paper was to focus on the debonding phenomenon between the FRCM reinforcement system and the concrete substrate, varying the artificial aging environment.

### 2.1. Mechanical Properties of Strengthening System

**Figure 1:**
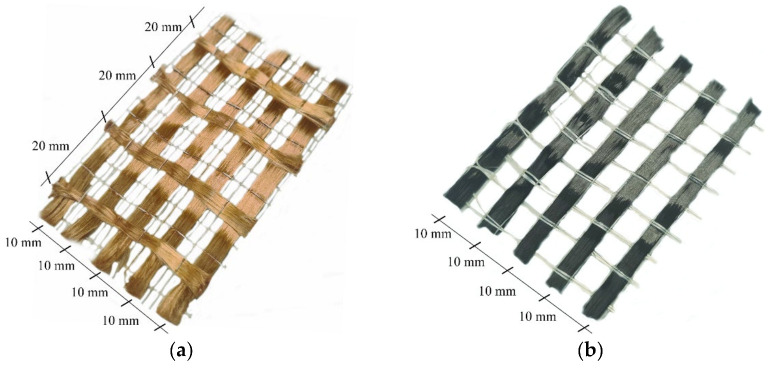
Geometry of dry fibers: (**a**) PBO Fibers and (**b**) C Fibers.

The mesh of the fabrics adopted in the FRCM system presents a development in mono-directional or bi-directional of fiber bundle and different weights depending on the type of element necessary in the strengthening technique (beam, column or wall). The strengthening system used in this study consists of fabric meshes embedded into cement-based mortar. Two type of fibers PBO and Carbon (C) were used. PBO-fibers presents the unbalanced fiber bundles along two orthogonal directions. In particular, the PBO-fiber bundles were spaced, respectively, 10 mm (longitudinal bundles) and 20 mm (transversal bundles), respectively, from the center (Figure 1a). The mass density of the fabric mesh was equal to 70 g/m^2^ (principal direction) and 18 g/m^2^ (transversal di-rection), while the equivalent thickness (t*) was equal to 0.045 mm (principal direction) and 0.012 mm (transversal direction). The C-fibers present a monodirectional fabric mesh, the single fiber bundles were spaced 10 mm from the center (Figure 1b). The mass density and equivalent thickness (t*) of the fiber bundles was equal to 182 g/m^2^ and 0.10 mm, respectively.

**Figure 2:**
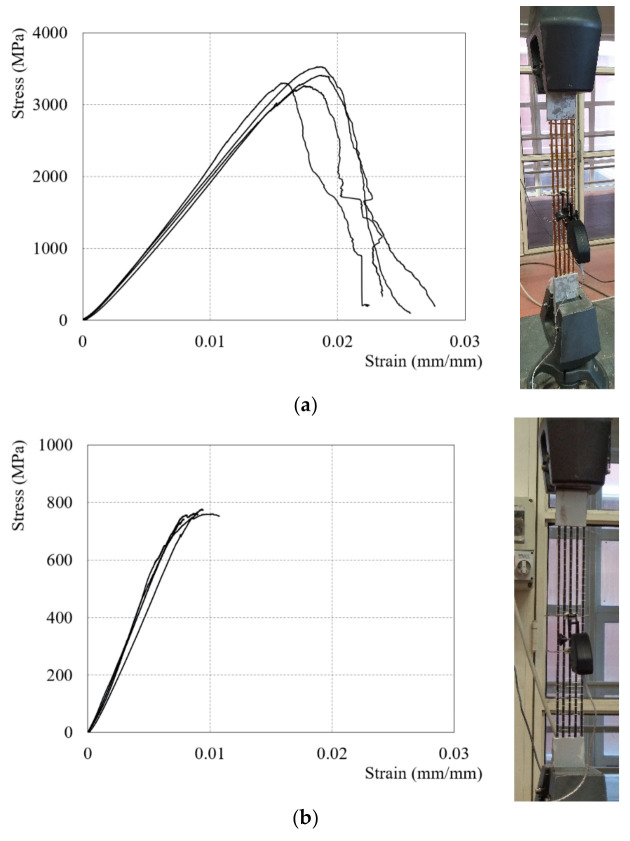
Tensile test on dry fiber: (**a**) PBO Fibers and (**b**) C Fibers.

Tensile strength, ultimate strain, and elastic modulus of the fibers were measured in accordance with [28]. Therefore, additional information on the fiber tensile tests is reported in [15,16,17]. Four coupons of both fiber types were tested. The fiber strips in accordance with [28] had 500 mm of length; moreover, at both specimens’ ends, two pairs of aluminum plates were attached by a thermosetting epoxy in order to guarantee a homogeneous constant pressure. At the center of the specimen, an extensometer, in order to evaluate the fiber strain with a gauge length of 50 mm, was installed. The tests, in displacement control at a rate of 0.5 mm/min, were conducted while both ends were clamped. In Figure 2, the test setup and the stress-strain diagram are reported, respectively, and, in Table 1, the obtained results from the dry fibers are summarized.

The composite systems adopted use a different cementitious mortar patented by the manufacturer [29].

The mortar is a stabilized, mono-component, inorganic matrix consisting of a special pozzolanic binder, selected aggregates, and special additives. The mechanical properties (compressive and flexural strength) in terms of average values and the coefficient of variation (CoV) are summarized in Table 2. The mortar prisms had a nominal dimension equal to 40 × 40 × 160 mm. In addition, the tests were conducted in load control in accordance with [30]. Finally, the mortar used with PBO and Carbon fibers were called M-PBO and M-C, respectively.

### 2.2. Specimen Preparation

**Figure 3:**
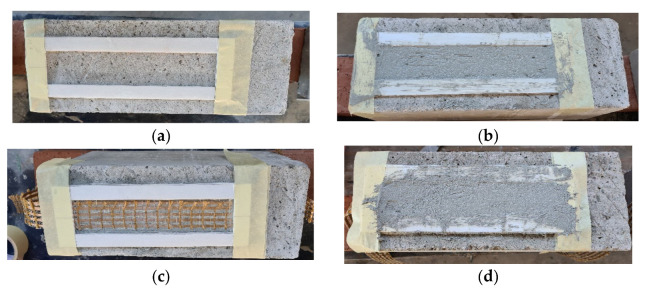
Specimen preparation strengthened with one layer: (**a**) Apply board panel, (**b**) apply internal layer, (**c**) PBO-fiber, and (**d**) apply external layer.

Casting of the concrete joints was made by ready-mix concrete with Portland cement (Type 1) without admixtures. All concrete joints had a rectangular cross section equal to 200 × 150 mm and a height of 400 mm. The compression and the tensile strength were equal to 26.1 MPa (CoV 0.03) and 2.4 MPa (0.05), respectively, in accordance with [31]. After the 28 days of curing, the lateral surfaces with a width of 150 and 200 mm were sandblasted to remove cement wastes and improve the gripping with the reinforcement system, as suggested by [29]. Before applying the strengthening system, the surface was made wet by cotton sheets. The technique employed in the application of the composite strips was organized into three steps. The first step consisted of adhesive tape attachment of a polystyrene board panel on the concrete surface with a thickness of 3 mm. A shape with a length and width of 260 mm (bonded length l) and 50 mm (width) was cut out of the board panel to accommodate the composite strips (Figure 3a). The composite strip was applied along the centerline of one face of the concrete prism at a time. Then, the mortar (called the internal layer) was applied by mold with light pressure to adhere the mortar to the concrete substrate (Figure 3b). In the second step, a fiber was placed on the internal layer and it was extended beyond the top of the board panel (Figure 3c). However, the second step is the most critical one, where the perfect alignment of the fibers and impregnation with the matrix must be ensured. Both types of fibers were composed of five yarns and the principal direction was positioned parallel to the bonded length. Finally, in the third step, the same operations as in step 1 were repeated (Figure 3d). Additionally, in this step, a slight pressure was applied so that the fibers were impregnated with mortar (called the external layer). The experimental campaign also included composite strips equipped with two layers of fibers. To make them, it was necessary, again, to perform the second and third steps. The composite strips equipped with two layers of fibers were composed of three layers of mortar and the second layer was called the intermediate layer. Each layer of mortar had a thickness of 3 mm. In all applications of the composite strips, the bonded area was started at 20 mm off the edge of the top face of the concrete prism. Four faces for each concrete prism were used, since the concrete joints were casted vertically from the top face. Moreover, the top face was troweled smooth and then made planar. The specimen’s curing was performed at 20 °C and 95% R.H. for 28 days.

### 2.3. Alkaline Environment Protocol (AK)

The alkaline environment adopted was reported in [28,32]. The alkaline solution suggested in [32] was made up of 1 liter of tap water with 118.5 of Ca(OH)2, 0.9 g of NaOH, and 4.2 g of KOH. The concrete joint specimens were completely immersed in a steel tank at a temperature of 23 °C ± 2 °C with a pH equal to 9.5. The steel tank was constituted of the steel cover, and all sides were made by two layers of steel with a thermal insulation in the middle in order to avoid the interaction with the atmosphere and maintain a constant temperature inside the tank. In addition, the steel tank was equipped with a double base so that electrical resistances did not touch the conditioned specimens. The specimens (concrete joint) were immersed in the solution for 1000 h, as suggested in [28,32]. During the conditioning time, the temperature and the pH solution were measured by means of a temperature sensor and pH-meter directly immersed into the solution. After the conditioning time, all specimens were immersed and cured in a tank with tap water for 28 days.

### 2.4. Hot Water Environment Protocol (HW)

Before conditioning the concrete joints’ and mortar prisms’ specimens, the water was preheated in a steel tank by an electric heater at the conditioning temperature of 40 °C ± 2 °C. The specimens were totally immersed and placed on a planar base. In addition, the specimens were positioned such that they did not touch one another and had the same level of water on top of them. The conditioning time was equal to 1000 and 3000 h. Periodically, the level, quality of the water, and the temperature inside of the steel tank were checked during the conditioning time. In particular, the standard [33] permits the replacement or addition of water to maintain the original standard.

### 2.5. Direct-Shear Test Protocol

**Figure 4:**
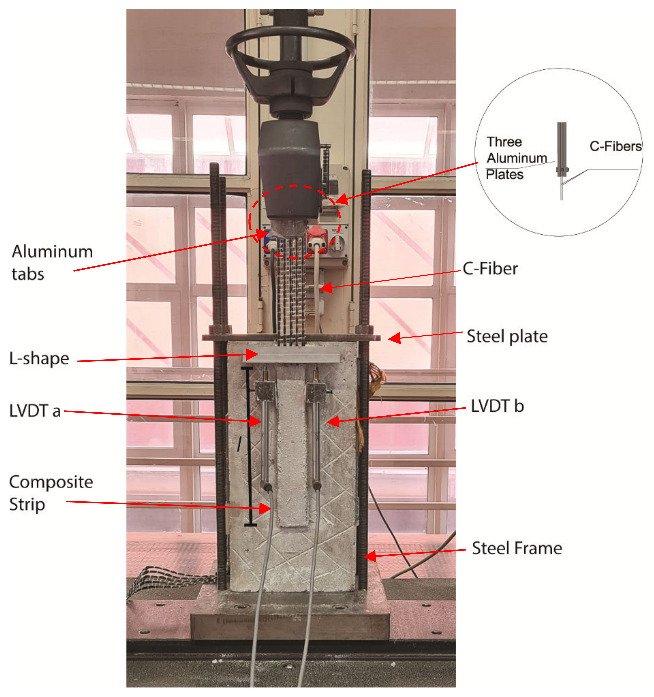
Test setup.

Forty-eight specimens of FRCM-concrete joints (control and subjected to conditioning) were tested by single-lap (direct) shear test setup using the push–pull configuration [10,11,12,13,14,15,16,17]. All specimens were named using the following notation DS-PBO (or C)-U (AK or HW)-ηL -µh (if present) -Z. DS indicates that the specimen was tested by single-lap, direct-shear. PBO or C indicates the type of fiber used. U (AK or HW) indicates the unconditioned specimen (U) or type of conditioning alkaline (AK) or hot water (HW). The ηL indicates the number of fiber layers. The µh indicates the time of the conditioning. Z = specimen number. The test setup used is shown in Figure 4, where the concrete joint was positioned under the steel frame. The latter was composed of a steel plate bolted to four steel bars bolted, in turn, to the machine used for the test. The shape of the steel frame was realized in order to restrain the movement of the concrete joint during the test and to allow the composite strip to be housed on sides of the prism not involved in the test. At the top of the composite strip, a couple of aluminum tabs were glued on the dry fiber by thermosetting epoxy to apply the load uniformly at the fiber bundles. For the composite strip equipped with two layers of fibers, an additional aluminum tab was used in order to maintain the fibers parallel to the applied load (Figure 4). During the test, the average value of the displacement of the dry fibers, called global slip *s* in technical literature, was applied. The global slip was evaluated by the reaction of an *L*-shaped aluminum plate attached to the dry fiber at the top near the bonded area by two linear variable displacement transformers (LVDTs), called LVDT a and b, glued at both sides of the bonded area. All tests reported in this work were conducted under displacement control using a universal testing machine. The rate adopted was 0.2 mm/min in accordance with [28]. Unconditioned specimens were tested at 28 days.

## 3. Results and Discussion

The results of the tests presented in this experimental study are reported in Table 3 in terms of peak load Peak Load P* and average value of P^*^_avg_ evaluated for each group of the specimens (unconditioned and conditioned). In addition, a parameter Δ* was evaluated for each group of specimens using the following Equation (1):

(1) $$\Delta^{*}=\frac{P_{avg\left(conditoned\right)}^{*}-P_{avg\left(unconditioned\right)}^{*}\;}{P_{avg\left(unconditioned\right)}^{*}\;}\;100\%$$

The failure observed for the specimens equipped with 1L and 2L was due to the debonding at the fiber-matrix interface with substantial slippage of the fibers, as reported in [10,15,16]. This phenomenon is called telescopic failure in the literature, where the outer filaments of each single bundle are impregnated with mortar while the internal filaments that constitute the core of the bundle presented a better ease of slippage due to the friction amongst the single filaments themselves. During the application of the load, individual filaments slipped at the interface between the two matrices and failure occurred progressively from the most external filaments to the core filaments at final collapse.

**Figure 5:**
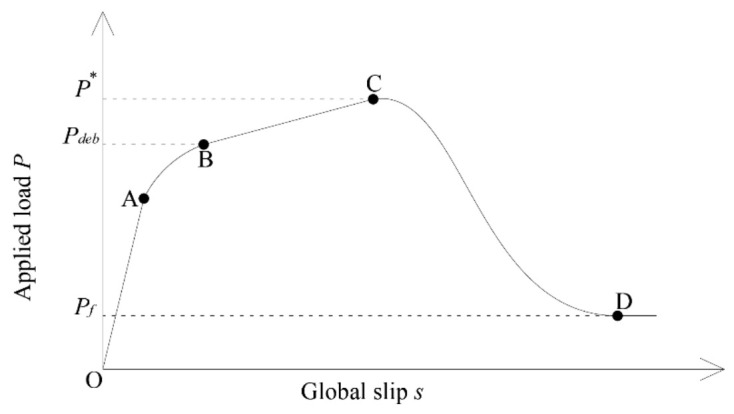
Idealized applied load *P*, global slip *s*.

The mortar and the bonding have the function of protection and load transfer to the substrate, respectively. The transfer occurs by contact (impregnation) between the two materials: fiber and mortar. The bonding capacity is directly proportional to the matrix penetrability coating and the friction between the fibers in each yarn. Figure 5 shows an idealized applied load-global slip response, proposed by D’Antino et al. [15]. The first part (OA) is a linear branch associated with elastic behavior of the bond between the PBO fiber net and the matrix. After point A, a non-linear branch (AB) follows until the load reaches *P_deb_* (point B). The non-linear branch is associated with micro-damage at the interface between fibers and matrix. At point B, the stress transfer zone (STZ) is fully established. The idealized *P*-*g* curve has a linear branch between points B and C that is dependent on the type of reinforcement and the extended bond length type, probably due to the friction (interlocking) between single-fiber filaments. At Point C, which corresponds to the peak load P*, the STZ reached the free end. After point C, the applied load decreased, due to the residual bonded length that was smaller than the length of the fully established STZ. The load response after point D was constant (*P_f_*).

**Figure 6:**
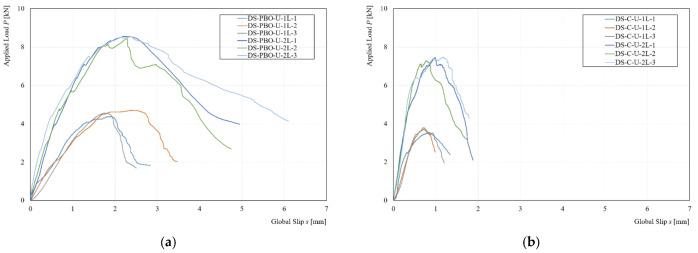
Experimental curve of unconditioned specimens: (**a**) PBO-FRCM and (**b**) C-FRCM.

Figure 6 shows the comparison between the experimental curves of the unconditioned specimens (Control).

From the comparison of the experimental curves, reported in Figure 6, it is possible to observe that in the first branch (O-A) the composite strips equipped with two layers of fibers (2L) presented an increase of initial stiffness. This phenomenon was more evident in the specimens strengthened with the PBO-fibers (PBO-FRCM) with respect to specimens equipped with C-fibers (C-FRCM). However, in terms of peak load, the strengthening systems equipped with PBO and C fibers achieved a P* of 54% and 49%, respectively.

### 3.1. Effect of Alkaline and Hot Water Environment

#### 3.1.1. Visual Inspection and Failure Mode

**Figure 7:**
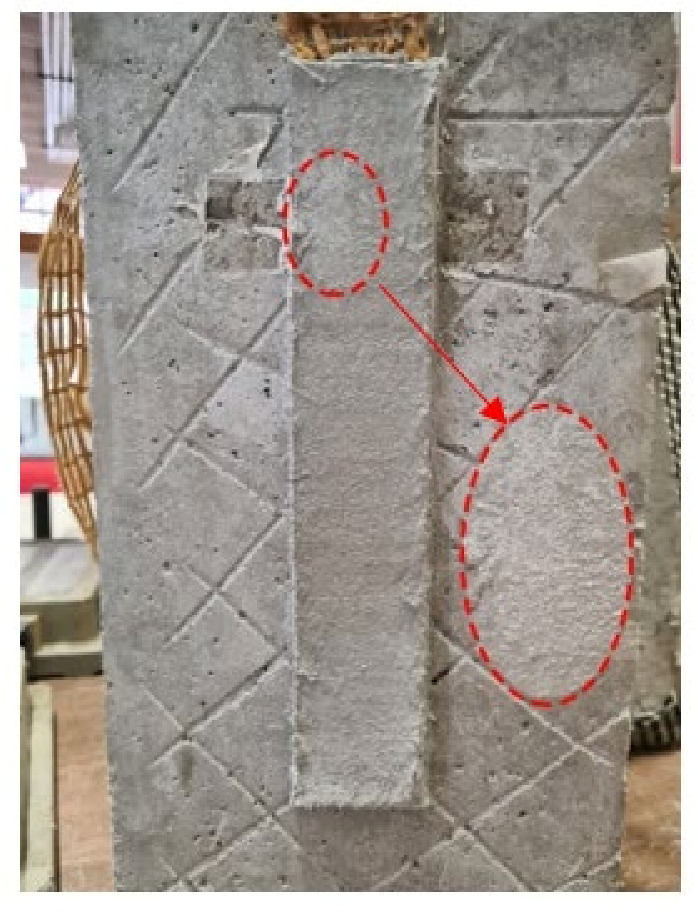
Visual inspection on specimen DS-PBO-AK-1L-2.

Specimens after 28 days of post-conditioning treatment were inspected visually with a 5× magnifying glass. The specimens did not show any damage. On the external surface of both the matrix and the dry fiber, located outside of the bonded area, an efflorescence appeared (Figure 7). This phenomenon was also observed in other studies [17,18,25].

**Figure 8:**
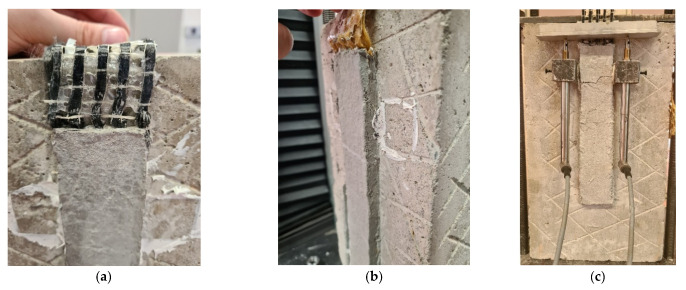
Failure mode observed in: (**a**) DS-C-HW-1L-1000h-2, (**b**) DS-PBO-HW-2L-3000h-3, and (**c**) DS-C-AK-2L-1000h-1.

A similar failure mode with respect to the unconditioned specimens was observed, where substantial slippage of the fibers at the mortar–fiber interface was independent of the type of conditioning. Figure 8 shows the slip at the loaded end of the specimen, in particular, for the specimen reinforced with a 1L (Figure 8a) and 2L (Figure 8b), respectively.

Only one specimen equipped with two layers of fibers presented a series of cracks on the external matrix during the test. The specimen after inspection did not present any damage due to alkaline conditioning and the cracks were probably due to misalignment of the fibers at the time of application of the load (Figure 8c).

#### 3.1.2. Applied Load *P*, Global Slip *s* Response

**Figure 9:**
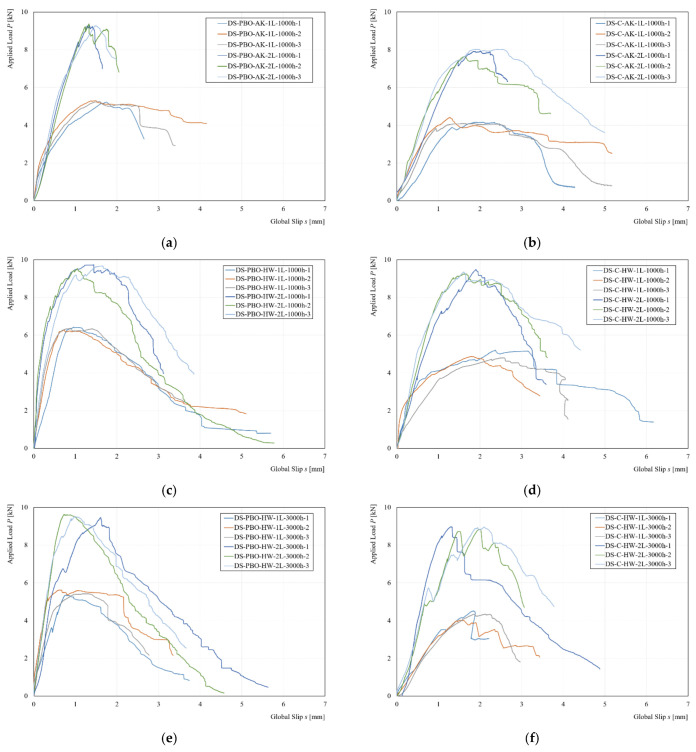
Comparison between experimental curve: (**a**) AK for PBO-FRCM, (**b**) AK for C-FRCM, (**c**) HW at 1000 h for PBO-FRCM, (**d**) HW at 1000 h for C-FRCM, (**e**) HW at 3000 h for PBO-FRCM. and (**f**) HW at 1000 h for C-FRCM.

Both reinforcement systems presented a light protection on the external filaments of dry fibers, as reported in [24,29], in order to preserve it from possible external agents but also from humidity and temperature variation during the curing phase of the mortar itself. Figure 9 shows the experimental applied load P versus global slip s response curves for each conditioned environment and type of reinforcement.

From a first analysis, it is possible to observe that the conditioned environment (alkaline and hot water) increased the capacity bond with increments in terms of peak load for both the strengthening systems. All conditioned specimens also showed an initial stiffness increment. The major increases were observed in the 1000-h hot water-conditioned environment in the specimens reinforced with 1L fiber layer with an increase between 37% to 35% for the specimens reinforced with PBO-FRCM and C-FRCM, respectively. For specimens reinforced with 2L layers of fibers, an increase was observed, but less if compared to specimens equipped with 1L, equal to 13 and 26 for PBO-FRCM and C-FRCM, respectively. Moreover, a similar increase in terms of peak load of 14% and 15% for specimens equipped with 1L of PBO and C-FRCM conditioned in alkaline environment was observed, while specimens equipped with 2L showed smaller increases of 9% and 6%. The increase of bond capacity should be attributed to an extended curing time at a temperature of 40 °C ± 2 °C and 23 °C ± 2 °C due to hot water- and alkaline-conditioned environments. Furthermore, the compatibility of the mortar with the substrate (concrete), high strength (mortar), and the low permeability of the materials (concrete and mortar) contributed to the increase of performance of the reinforcement [20,21,22,23,24,25]. However, with a greater conditioning time of 3000 h, it was possible to note a decrease in terms of peak load compared to the specimens conditioned with a lesser time. However, it was possible to notice a different trend with the specimens equipped with 2L, in which they exhibited a lower reduction from 13% to 12% and 26% to 21% for the C-FRCM and PBO-FRCM systems. This phenomenon was caused by more protection of the reinforcement, in particular for the most internal fiber layer. Furthermore, in the specimens conditioned with hot water, the experimental curves presented a series of load drops for the entire applied load response; the phenomenon is more visible on specimens with a conditioning time of 3000 h. Specimens conditioned in an alkaline environment showed smaller increases than samples conditioned in hot water for the same conditioning time, independent of reinforcement type and number of reinforcement layers. The temperature difference of the conditioned environment may have affected the longer curing period of the conditioned specimens. In addition, it was possible to see a tendency of the load friction less than that of unconditioned specimens. The alteration of the experimental curves should be attributed to a modification of the matrix fiber interaction for the more external filaments and between the friction between internal filaments of the core. Both systems showed an excellent resistance to alkaline and hot water environments [21,22,24,25]. The presence of a protective film and the type of matrix plays a fundamental role in the durability of the reinforcement system.

## 4. Conclusions

Durability is a very important factor for the service life of concrete or masonry structures. The paper presented the experimental investigation on the effects of alkaline and hot water environments on the FRCM composite system using two different types of fibers: PBO and Carbon, respectively. The investigated parameters were the number of reinforcement layers, FRCM reinforcement type, time, and conditioning type. Based on the results, the following conclusions were made:

- The peak load P* of the specimens with two layers of dry fiber nets was roughly double with respect to the specimens with one layer of dry fiber nets, independent of the strengthening system considered;
- Both systems exhibited an excellent resistance to alkaline and hot water environments;
- Protective film and the type of mortar plays a fundamental role in the durability of the reinforcement system;
- On the external surface of both the matrix and the dry fiber outside of the bonded area, efflorescence appeared for the specimens conditioned in alkaline environment;
- Conditioned specimens, independently of the conditioning type, showed an increase in initial stiffness;
- Specimens conditioned at 1000 h in a hot-water treatment showed a major increase in terms of applied peak load;
- A longer conditioning time could alter the fiber–matrix interaction and the interaction between the fibers themselves;
- Specimens equipped with 2L fiber layers exhibited greater preservation of their bond capacity than specimens strengthened with 1L fiber layer.

Besides this preliminary work, further studies are necessary to understand the stress-transfer mechanism with more exposure time in the alkaline environment; combination of the alkaline environment with an additional environment agent, for example, hot water; and different types of matrices with different mechanical performances.

## Figures and Tables

**Table 1:** Mechanical properties of the PBO and Carbon Fibers.

	PBO	Carbon
Tensile strength [GPa] (CoV)	3.40 (0.04)	0.78 (0.03)
Ultimate strain [%] (CoV)	2.5 (0.08)	1.1 (0.27)
Elastic modulus [GPa] (CoV)	214 (0.04)	112 (0.07)

**Table 2:** Mechanical properties of the matrix.

	M-PBO	M-C
Flexural tensile strength [MPa] (CoV)	7.08 (0.14)	7.77 (0.09)
Compressive strength [MPa] (CoV)	44.20 (0.08)	45.47 (0.05)

**Table 3:** Experimental results.

Specimen	P* [kN]	P*_avg_ [kN]	Δ* [%]
DS-PBO-U-1L-1	4.60	4.62	
DS-PBO-U-1L-2	4.70		-
DS-PBO-U-1L-3	4.56		
DS-PBO-U-2L-1	8.55	8.51	
DS-PBO-U-2L-2	8.44		-
DS-PBO-U-2L-3	8.54		
DS-C-U-1L-1	3.52	3.67	
DS-C-U-1L-2	3.70		-
DS-C-U-1L-3	3.79		
DS-C-U-2L-1	7.45	7.40	
DS-C-U-2L-2	7.28		-
DS-C-U-2L-1	7.47		
DS-PBO-AK-1L-1000h-1	5.22	5.26	
DS-PBO-AK-1L-1000h-2	5.30		14
DS-PBO-AK-1L-1000h-3	5.25		
DS-PBO-AK-2L-1000h-1	9.27	9.31	
DS-PBO-AK-2L-1000h-2	9.37		9
DS-PBO-AK-2L-1000h-3	9.29		
DS-C-AK-1L-1000h-1	4.16	4.22	
DS-C-AK-1L-1000h-2	4.41		15
DS-C-AK-1L-1000h-3	4.09		
DS-C-AK-2L-1000h-1	7.92	7.86	
DS-C-AK-2L-1000h-2	7.63		6
DS-C-AK-2L-1000h-3	8.03		
DS-PBO-HW-1L-1000h-1	6.43	6.35	
DS-PBO-HW-1L-1000h-2	6.27		37
DS-PBO-HW-1L-1000h-3	6.35		
DS-PBO-HW-2L-1000h-1	9.74	9.65	
DS-PBO-HW-2L-1000h-2	9.53		13
DS-PBO-HW-2L-1000h-3	9.67		
DS-C-HW-1L-1000h-1	5.21	4.96	
DS-C-HW-1L-1000h-2	4.87		35
DS-C-HW-1L-1000h-3	4.80		
DS-C-HW-2L-1000h-1	9.48	9.36	
DS-C-HW-2L-1000h-2	9.25		26
DS-C-HW-2L-1000h-3	9.35		
DS-PBO-HW-1L-3000h-1	5.34	5.46	
DS-PBO-HW-1L-3000h-2	5.62		18
DS-PBO-HW-1L-3000h-3	5.43		
DS-PBO-HW-2L-3000h-1	9.47	9.54	
DS-PBO-HW-2L-3000h-2	9.63		12
DS-PBO-HW-2L-3000h-3	9.51		
DS-C-HW-1L-3000h-1	4.51		
DS-C-HW-1L-3000h-2	4.03	4.30	17
DS-C-HW-1L-3000h-3	4.35		
DS-C-HW-2L-3000h-1	8.97		
DS-C-HW-2L-3000h-2	8.83	8.92	21
DS-C-HW-2L-3000h-3	8.95		

## Data Availability

The data presented in this study are available on request from the corresponding author.
